# Hawthorn (*Crataegus* spp.) Clinically Significantly Reduces Blood Pressure in Hypertension: A Meta-Analysis of Randomized Placebo-Controlled Clinical Trials

**DOI:** 10.3390/ph18071027

**Published:** 2025-07-10

**Authors:** Zsóka Szikora, Rebeka Olga Mátyus, Bettina Vargáné Szabó, Dezső Csupor, Barbara Tóth

**Affiliations:** 1 Institute of Clinical Pharmacy, Faculty of Pharmacy, University of Szeged, 6725 Szeged, Hungary; szikora.zsoka@szte.hu (Z.S.); matyus.rebeka@szte.hu (R.O.M.); bettina.szabo.szb@gmail.com (B.V.S.); 2 Institute of Translational Medicine, University of Pécs, 7624 Pécs, Hungary

**Keywords:** *Crataegus*, hawthorn, hypertension, meta-analysis, antihypertensive

## Abstract

**Background/Objectives**: Hypertension affects over 1.3 billion people globally, and inadequate therapy is reported in 80% of cases. Patients increasingly turn to complementary therapies, including hawthorn (*Crataegus* spp.), a traditional remedy for cardiovascular diseases. Hawthorn has long been used in folk medicine to lower blood pressure; however, its efficacy has not been fully established. This meta-analysis aimed to evaluate the antihypertensive effects and safety of hawthorn in randomized, placebo-controlled trials. **Methods**: A systematic review and meta-analysis were conducted, including six studies with a total of 428 participants. The trials focused on systolic (SBP) and diastolic blood pressure (DBP) changes over treatment periods of 10 weeks to 6 months. Literature searches were conducted in the Embase, PubMed, Cochrane, and Web of Science databases. Studies that met the predefined PICO criteria were included. Data analysis was performed using a random-effects model, and the risk of bias was assessed using the Cochrane Risk of Bias Tool. **Results**: Hawthorn statistically significantly decreased SBP (MD: −6.65 mmHg; 95% CI [−11.72; 1.59]) and non-significantly reduced DBP (MD: −7.19 mmHg; 95% CI [−15.17; 0.79]) after 2–6 months of treatment. Variations in dosage (250–1200 mg/day) and study protocols contributed to this heterogeneity. **Conclusions**: The effect of hawthorn on blood pressure is clinically significant. However, larger, well-designed trials are needed to establish optimal dosing, duration, and efficacy with greater reliability.

## 1. Introduction

During the last three decades, the number of hypertensive patients has doubled: in 1990, approximately 650 million adults had hypertension, and the number of hypertensive patients reached 1.3 billion by 2019. According to an estimate by the WHO in 2023, one in three adults has hypertension, but half of the patients are not diagnosed with this condition [1]. Several types of antihypertensive agents are available for the treatment of hypertension in adults, including angiotensin-converting enzyme (ACE) inhibitors, angiotensin II receptor blockers, calcium channel blockers, and diuretics [2]. Despite the availability of many therapeutic options to reduce blood pressure, it is estimated that therapy for four out of five hypertensive patients is inadequate, mainly due to reduced adherence and poor compliance [1].

It is common for patients to seek more affordable and presumably safer alternatives to replace or complement their pharmacotherapy; therefore, the popularity of complementary and alternative medicine (CAM) has been steadily increasing [3,4].

Hawthorn (*Crataegus* spp.), a member of the Rosaceae family, has been used for medicinal purposes since ancient times [5]. Hawthorn fruit has potent antioxidant and free radical scavenging properties due to the presence of several bioactive compounds, such as flavonoids (hyperoside and quercetin) and oligomeric proanthocyanidins (epicatechin and procyanidin) [4,6,7]. Its protective effects on the cardiovascular system have also been thoroughly studied in in vitro, in animals, and in humans [8,9]. Hawthorn leaf and flower (*Crataegi* folium cum flore) can be used as a traditional herbal medicinal product to relieve symptoms of temporary nervous cardiac complaints (e.g., palpitations, perceived extra heartbeats due to mild anxiety) after serious conditions have been excluded by a medical doctor [10]. It has been suggested as an alternative therapy for several cardiovascular conditions, such as angina pectoris, hypertension, hyperlipidemia, cardiac dysrhythmias, and New York Heart Association (NYHA) functional class II congestive heart failure [11,12]. Several studies have indicated that hawthorn affects blood pressure. A recent meta-analysis revealed that the combination of hawthorn and d-camphor has antihypotensive effects; however, when applied as monocomponent products, hawthorn had antihypertensive effects [13]. Although several randomized, placebo-controlled clinical trials have been conducted to evaluate the effects of hawthorn on blood pressure, the data have not yet been statistically synthesized in a meta-analysis. A recently published systematic review found that hawthorn can reduce blood pressure; however, the results of the individual trials included in this review were not statistically analyzed [14]. Therefore, to evaluate the antihypertensive effects of *Crataegus* species based on the available published evidence, a literature review and meta-analysis were conducted. The present investigation was designed to assess the efficacy and safety profile of hawthorn in human clinical trials.

## 2. Results

### 2.1. Literature Search and Study Selection

**Figure 1:**
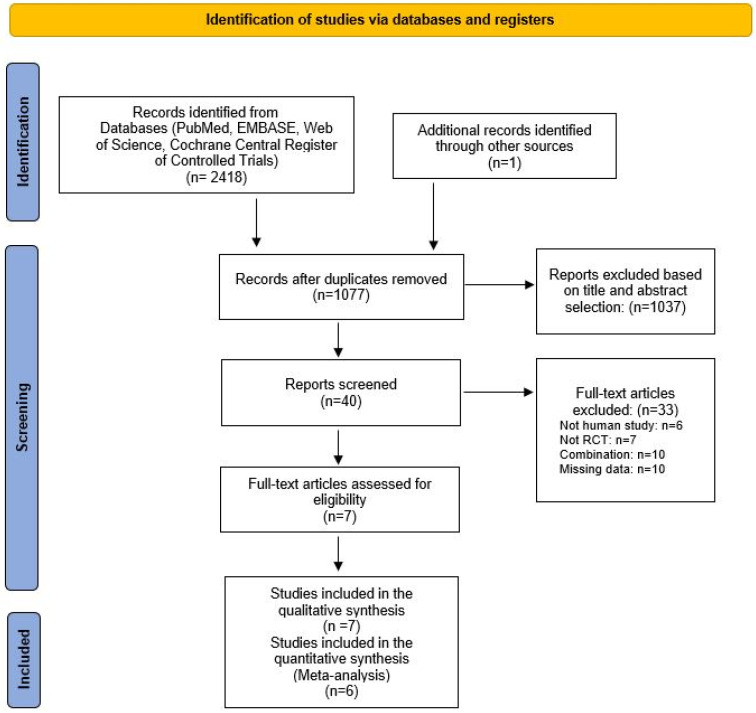
PRISMA flow diagram for identifying relevant studies.

A literature search was conducted using the search terms “crataegus” and “hypertension” in the Embase, PubMed, Cochrane Central Register of Controlled Trials, and Web of Science databases. After removing duplicates, the search yielded a total of 1073 potentially relevant reports. An additional report [15] was identified by selecting the reference lists from a previous review on this topic (Review: The effect of hawthorn on blood pressure) [14]. RCTs to be included in the meta-analysis were selected according to the flow chart presented below (Figure 1).

After a detailed review of the abstracts, 40 RCTs were selected for full-text screening. Several articles were excluded after reading the full text due to incomplete data or non-compliance with our PICO (e.g., hawthorn was combined with other drugs). Seven studies with 449 patients were selected for qualitative analysis. Of the 449 participants, 218 were men and 139 were women. One trial did not report the sex of the participants (n = 92). All studies included adult patients with high blood pressure (SBP: >140 mmHg; DBP: >90 mmHg). One study involved patients with hypertension and sleep disorders, while another involved patients with type 2 diabetes mellitus. Of these seven studies, one RCT [16] was excluded from the statistical analysis. In this crossover study of four periods, the administration period was 3.5 days; therefore, it evaluated the acute effects of hawthorn. It was not possible to compare the results of this study with those of other clinical trials measuring the long-term effects of hawthorn.

Finally, statistical analysis was performed on the data from six RCTs (n = 428). All articles included in the meta-analysis were randomized, placebo-controlled studies. The study drug was *Crataegus* spp. in all the cases. The articles were published between 2002 and 2021. The main characteristics of the included RCTs are presented in Table 1.

### 2.2. Risk of Bias Assessment

**Figure 2:**
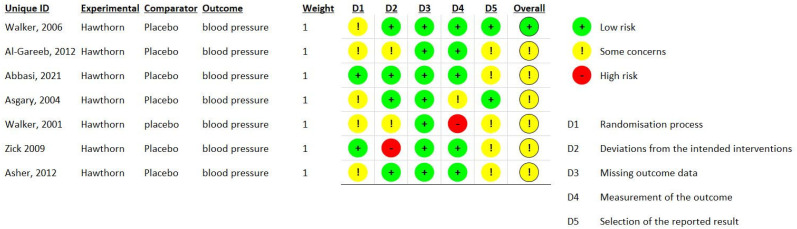
Summary of the risk of bias in the included studies (Green: Green indicates a low risk of bias; Yellow: Yellow indicates some concerns; Red: Red indicates a high risk of bias; Unique ID: identifier for each individual study, First author, the date of the publication; Experimental: the intervention under investigation; Comparator: the control treatment used for comparison; Outcome: the measured effect; Weight: the statistical weight assigned to each study in the meta-analysis, reflecting its contribution to the overall result.) [15,16,17,18,19,20,21].

Each study was evaluated for potential bias using the Cochrane Collaboration Risk of Bias Tool. For each domain, the trials were evaluated as having a high (red), unclear (yellow), or low (green) risk of bias (Figure 2).

One study showed a low risk of bias [19], while the other six had some concerns. These trials did not clearly report the method used for randomization; therefore, these RCTs were considered to have an unclear risk of selection bias. Studies that did not describe the methods used for randomization, allocation, or blinding were considered to have an unclear risk of selection, performance bias, or detection bias. One study [17] was judged to have a high risk of measuring the outcome, and another [20] had a high risk of deviations from the intended interventions. All studies showed concerns regarding reporting bias and an unclear risk of other types of bias.

### 2.3. Study Characteristics

The included randomized, placebo-controlled clinical trials were conducted in four different countries [Iraq (n = 1), the UK (n = 2), Iran (n = 2), and the USA (n = 1)] from 2002 to 2021. The key characteristics are summarized in Table 1. The sample sizes ranged from 17 to 120 patients. All species of *Crataegus* were included in the meta-analysis.

In five trials, powdered herbal substances (from leaves and flowers) were used, and in one study, hawthorn’s ethanol extract (DER 1:8) was used. In two RCTs [17,21], the participants received 500 mg of *Crataegus* powder daily; in two other studies, 900 mg of *Crataegus* powder was administered daily, and in one study, 1200 mg of *Crataegus* powder was used daily, and the liquid preparation contained 250 mg of *Crataegus* per day.

Two trials lasted 4 months [18,19], while 1-1 other trial lasted 2 [21] and 3 months [15], one trial lasted 10 weeks [17], and the longest study collected data for 6 months [20].

In four of the studies included in our meta-analysis, taking prescription antihypertensives or other medicines was an exclusion criterion [15,17,18,21]. In one study, participants were permitted to continue their previously prescribed therapy, which was recorded, and any changes made during the study were also documented [19]. In one trial, hawthorn or a placebo was used alongside well-established treatments for heart failure [20].

### 2.4. Patient Characteristics

The blood pressure values of 428 patients were statistically analyzed. The mean age of the patients was 53.98 years, 44.78% of the population were female, and 55.22% were male. Both women and men are affected by hypertension, with a ratio of 1.1:1 to 1.2:1 according to international data [22]. The study population was almost identical to that rate. All participants had elevated baseline blood pressure values, and the presence of comorbidities such as type 2 diabetes mellitus, sleep disorders, and heart failure was not an exclusion factor.

### 2.5. Outcome

The efficacy of *Crataegus* compared to a placebo was evaluated based on changes in blood pressure values. The results regarding changes in blood pressure are listed in Table 2.

**Figure 3:**
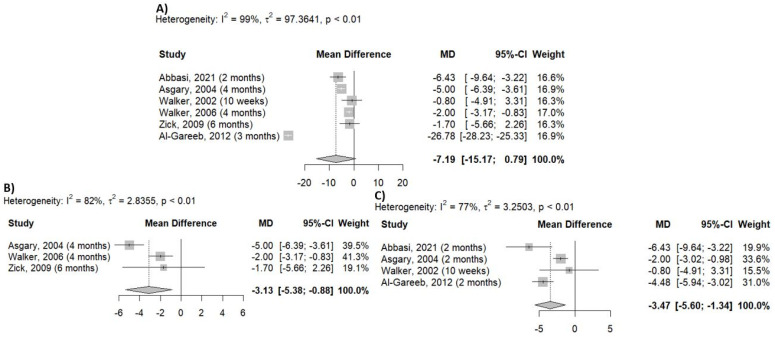
Effect of *Crataegus* on diastolic blood pressure based on the analysis of the whole dataset (**A**), the analysis of 4–6 months of data (**B**), and 10 weeks–2 months of data (**C**). (Mean difference: mean difference between placebo and *Crataegus*, MD: mean difference value, 95%-CI: 95% confidence interval, Weight: weight of the individual study; Random effects model: results obtained using the random effects model, and expressed as MD, 95% CI.) [15,17,18,19,20,21].

The results of RCTs on the antihypertensive effect of *Crataegus* versus placebo were examined separately for systolic and diastolic blood pressure values based on six randomized controlled trials (RCTs) [15,17,18,19,20,21]. A reduction in diastolic blood pressure (DBP) was observed after analyzing the whole dataset (MD = −7.19 mmHg; 95% CI [−15.17; 0.79]) (Figure 3A); however, the effect was not statistically significant from that of the placebo. If a trial measured the blood pressure-lowering effect at more than one time point, the effect of the longest treatment duration was considered in this analysis. When studies with different durations were analyzed separately, there was a significant reduction in the diastolic blood pressure, regardless of the duration. Statistically significant efficacy was observed in studies lasting for 4–6 months (MD = −3.13; 95% CI [−5.38; −0.88] (Figure 3B) and when *Crataegus* was used for 10 weeks–2 months (MD = −3.47 mmHg; 95% CI [−5.60; −1.34]) (Figure 3C).

**Figure 4:**
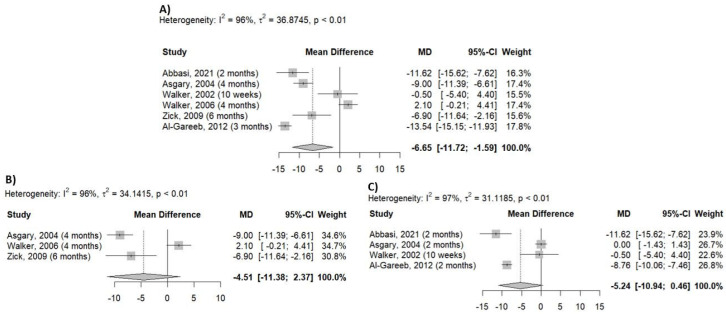
Effect of *Crataegus* on systolic blood pressure based on the analysis of the whole dataset (**A**), the analysis of 4–6 months of data (**B**), and the assessment of 10 weeks–2 months of data (**C**). (Mean difference: mean difference between placebo and *Crataegus*, MD: mean difference value, 95%-CI: 95% confidence interval, Weight: weight of the individual study; Random effects model: results obtained using the random effects model, and expressed as MD, 95% CI.) [15,17,18,19,20,21].

When data on systolic blood pressure (SBP) were analyzed, it was found that *Crataegus* decreased SBP not only clinically significantly, but this effect was also statistically different from that of placebo (MD = −6.65 mmHg; 95% CI [−11.72; −1.59] (Figure 4A). We performed a subgroup analysis based on the duration of the interventions, and the results were not significant. The lowest reduction in SBP was observed in studies that investigated the antihypertensive effects of *Crataegus* over the longest period (4–6 months) (MD, −4.51 mmHg; 95% CI [−11.38, 2.37]). (Figure 4B) A greater efficacy was observed in trials lasting between 10 weeks and 2 months (MD = −5.24 mmHg, 95% CI [−10.94; 0.46]) (Figure 4C).

The study with the four-period crossover design investigated the effect of hawthorn extract on flow-mediated dilatation (FMD) of the brachial artery in prehypertensive or mildly hypertensive adults. Participants were randomized to receive doses of hawthorn extract (1000 mg, 1500 mg, and 2500 mg) or placebo twice daily for 3.5 days, followed by FMD measurement and a washout period of 4 days between treatments. No relationship was found between the dose administered and the effects on FMD, brachial artery diameter, or blood pressure. Due to the different endpoints, this study was not included in the quantitative analysis of the meta-analysis [16].

Adverse reactions reported in the studies were documented. The most common adverse effects were headaches and mild gastrointestinal symptoms, such as nausea, which were reported by patients using hawthorn preparations. These symptoms were expected based on the previous literature and were the most common adverse reactions to *Crataegus* species [23].

## 3. Discussion

Hawthorn species have long been used in European folk medicine. Different plant parts and extracts made from them have been used for centuries for various indications in many countries in Europe, but one of the most prominent indications is as an antihypertensive [24]. Although its traditional use is recognized by the European Medicines Agency, the official monograph on the plant does not include hypertension as an indication (partly due to the lack of evidence of efficacy) [10]. The efficacy of hawthorn (*Crataegus* spp.) has been investigated mostly in the treatment of heart failure [18]. A previous meta-analysis [13] assessed the antihypertensive effect of *Crataegus* in combination with d-camphor. When used as a monotherapy, however, there is no evidence to suggest that hawthorn raises blood pressure; in fact, experience in folk medicine and preclinical data suggest the opposite [25]. Hawthorn may reduce blood pressure in several ways. It regulates bile acid metabolism, reduces inflammation, and improves endothelial function. The active principles of the plant, in particular flavonoids and proanthocyanidins, have a vasodilating effect, which reduces peripheral vascular resistance and blood pressure. They play a significant role in stimulating the release of nitric oxide (NO) produced by endothelial cells, a potent vascular smooth muscle relaxant that plays a key role in regulating vascular tone [26]. Hawthorn also improves oxygen supply to the myocardium and increases its contractile force, improving the pumping function of the heart. Its antioxidant properties protect blood vessels from oxidative stress and help maintain a healthy endothelium [27]. Some of its active ingredients also have ACE (angiotensin-converting enzyme inhibitor) activity, affecting the renin-angiotensin system, which plays an important role in blood pressure regulation. It also has a mild sedative effect that reduces the increase in blood pressure caused by stress [28]. Several recent clinical studies have shown that the use of an antihypertensive agent can be justified based on scientific evidence. This meta-analysis was designed to synthesize the available evidence for this use. Based on our most up-to-date meta-analysis, hawthorn (*Crataegus* spp.) is a promising agent for the treatment of hypertension. *Crataegus* used for 10 weeks–2 months and 4–6 months had statistically significant effects on diastolic blood pressure; however, the analysis of the whole dataset revealed statistically non-significant efficacy. Systolic blood pressure was significantly reduced based on the analysis of all studies, but the subgroup analysis resulted in a statistically non-significant efficacy. However, the observed effects were clinically significant. A meta-analysis of 208 clinical trials involving 94,305 people found that first-line antihypertensive monotherapy treatment reduced systolic blood pressure by 10–15 mmHg and diastolic blood pressure by 8–10 mmHg [29]. Our meta-analysis concluded that the maximum reduction achievable was 6.65 mmHg for SBP and 7.19 mmHg for DBP, which is comparable to the effect of first-line antihypertensives. Hawthorn is considered safe and has not been shown to interact with other medicines; therefore, it can be used to complement standard therapy [30]. This possibility was examined earlier in a single-blind controlled trial that assessed the effect of *Crataegus oxyacantha* as an adjunct to standard antihypertensive therapy, with the control group receiving standard antihypertensive therapy alone. In this study, a significant blood pressure-lowering effect was shown after 6 weeks of additive *Crataegus* treatment, but at other time points, hawthorn did not enhance the effect [31]. The limitations of our meta-analysis are closely related to the studies analyzed. In several trials, patients with comorbidities (diabetes mellitus) were included, resulting in a heterogeneous patient population for this meta-analysis. It is reasonable to assume that the inhomogeneous patient population, differences in the durations of treatments, and the characteristics of the applied hawthorn products may have influenced efficacy. The doses applied in the trials were also different. One trial used a 250 mg preparation as a drop [18], one trial used a 500 mg capsule once daily [17], and another used 250 mg capsules twice daily [21]. Two trials used a preparation containing 450 mg of *Crataegus* twice daily, in one trial as a capsule [15] and in another as a tablet, Crategutt Forte [20]. In one trial, patients took two 600 mg tablets [19]. An additional limitation is that crucial information regarding the products used was missing from almost every study, such as the exact species, plant part, time of collection, extraction conditions, extraction ratio, extraction time, and exact composition of the extracts. In summary, our results suggest that *Crataegus* reduces blood pressure and may be used as a complementary therapy for hypertension; however, the optimal dose and duration of treatment are not yet clear. Given these limitations, a cautious conclusion is that further and larger trials, conducted by independent research groups and using standard endpoints, are needed to assess the efficacy of *Crataegus* with a lower risk of bias. On the other hand, clinical studies evaluating herbal medicinal products should also describe the plant material more thoroughly. In 2006, the herbal extension of the CONSORT checklist was published. This recommendation defines the key elements that should be mentioned in publications reporting clinical trials of herbal medicinal products. These elements include the Latin binomial name of the herb, including the botanical authority and plant family, the specific name of the product, including the brand name, extract name (if applicable), and the manufacturer, the part(s) of the plant used to produce the preparation, the extraction method and the type of solvent used (e.g., 70% ethanol, water), including the drug-to-extract ratio, the dosage regimen and how it was determined, the dosage regimen and how it was determined, the nature of the control or placebo, including how it matches the herbal product in appearance, taste, and smell, the training and qualifications of practitioners involved in administering the intervention [32]. As we have seen in this meta-analysis, authors rarely adhere to these recommendations.

Our meta-analysis shows that although there is a tendency for *Crataegus* to have an antihypertensive effect, the efficacy is not sufficient to consider it as a monotherapy for the treatment of hypertension. Although it is theoretically plausible that *Crataegus* may improve the efficacy of antihypertensive treatment, further studies are needed to clarify the efficacy and safety of this therapy.

## 4. Materials and Methods

The meta-analysis was reported according to the PRISMA protocol and was registered in the International Prospective Register of Systematic Reviews (PROSPERO) on 13 June 2024 (registration ID: CRD42024557170).

The following PICO (patients, intervention, comparison, outcome, study design) format was applied: P: patients with hypertension, I: hawthorn extract per os, C: placebo, and O: systolic and diastolic blood pressure, S: randomized, placebo-controlled trials.

### 4.1. Information Sources and Search Strategy

Literature searches were conducted through the Embase, PubMed, Cochrane Central Register of Controlled Trials, and Web of Science databases until 1 August 2024, using the following search strategy: [(hawthorn) or (crataegus) or (crataegus spp.) or (thornapple)] and [(hypertension) or (hypotensive effect for high blood pressure) or (hypotensive effect for hypertension) or (antihypertensive) or (increase hypotension) or (decrease hypertension) or (reduce blood pressure)].

No language restrictions, publication dates, or publication statuses were applied. The reference lists of all the identified articles were inspected. Only publicly available data were analyzed, and neither the authors nor the manufacturers were contacted for additional information.

### 4.2. Eligibility Criteria and Study Selection

Randomized, placebo-controlled trials evaluating the effects of *Crataegus* spp. in adult patients with hypertension were included. Zotero 7.0 was used for reference management. After removing duplicates, the remaining records were screened for eligibility based on their titles and abstracts. The eligibility of the full texts of the resulting records was independently assessed by two reviewers (Z.S. and R.O.M.). In the case of disagreement between reviewers, a third reviewer (B.T.) was consulted.

### 4.3. Data Extraction and Synthesis of the Results

Data collection was performed according to the PRISMA guidelines. The characteristics and results of the study were extracted independently by the two reviewers (Z.S., R.O.M.) Discrepancies in the extracted data were resolved through discussion. The following data elements were extracted from the included articles: study design, characteristics of the patient population and sample size, intervention details, type of comparator(s), outcome measures, and overall results. Systolic and diastolic blood pressure values were extracted as the outcome measures. Discrepancies in the extracted data were resolved through discussion between the two reviewers.

### 4.4. Risk of Bias Analysis

The risk of bias was analyzed using the Cochrane Risk of Bias Tool (version 2.0). Two authors independently completed the bias risk assessment, and any differences were resolved by consensus. If the risk of bias was low in all domains, the overall risk of bias in each trial was considered low; if the risk of bias was high in at least one domain, the overall risk of bias in each trial was considered high. In any other context, the risk of bias was considered to be a concern.

### 4.5. Statistical Analysis

Statistical analysis was performed using RStudio software (version 2024.9.0.375) and the meta package. To compare the mean data, Mean Difference (MD) with 95% CIs were computed for systolic and diastolic blood pressure. The results are presented in forest plots (Figure 2 and Figure 3). Heterogeneity was examined using the Higgins I^2^ indicator, which was the percentage of total variation [33]. According to the Cochrane Handbook, I^2^ can be low (0–40%), moderate (30–60%), substantial (50–90%), or considerable heterogeneity (75–100%).

A random-effects model was used to conduct the meta-analysis. This model assumes that the true effect size can differ across studies. In this case, the summary effect is an estimate of the mean of the distribution of true effects. The weights of the studies were based on the within-study variance plus τ^2^, which is the between-study variance. The τ^2^ reduces the relative differences among weights. This means that large studies lose their influence, and small studies gain influence [34].

## 5. Conclusions

Based on this meta-analysis, *Crataegus* spp. (Hawthorn) has demonstrated clinically significant efficacy in reducing blood pressure compared with placebo. The reduction in systolic blood pressure was statistically significant; however, the efficacy in the case of diastolic blood pressure remained non-significant. Although these results are promising, larger, well-designed, large-scale trials are needed to define the optimal dose and duration of treatment and address heterogeneity in patient populations.

## Figures and Tables

**Table 1:** Characteristics of the studies (all placebo-controlled, randomized trials) included in the final quantitative analysis.

First Author (Year)	Country	Group	Dose	Duration	Patient Characteristics	Outcome Measure
Ann F. Walker (2002) [17]	UK	*Crataegus* spp. Placebo	500 mg capsule /daily	10 weeks	mild hypertension (DBP: 85–100 mmHg)	Changes in blood pressure
Asgary S. (2004) [18]	Iran	*Crataegus curvisepala* Placebo	250 mg drops /daily	4 months	mild hypertension (SBP: 140–159 mmHg; DBP: 90–95 mmHg)	Changes in blood pressure
Ann F. Walker (2006) [19]	UK	*Crataegus laevigata* Placebo	600 mg tablet/twice daily	4 months	hypertension (SBP: 145–165 mmHg; DBP: 85–95 mmHg) type 2 diabetes mellitus	Changes in blood pressure
Suzanna M. Zick (2009) [20]	USA	*Crataegus* spp. Placebo	450 mg Crataegutt forte tablet /twice daily	6 months	NYHA class II–III chronic heart failure	Changes in blood pressure
Al-Gareeb (2012) [15]	Iraq	*Crataegus* spp. Placebo	450 mg capsule/twice daily	3 months	first stage of hypertension (SBP: 140–159 mmHg; DBP: 90–99 mmHg)	Changes in blood pressure
Masumeh Abbasi (2021) [21]	Iran	*Crataegus monogyna* Placebo	250 mg capsule/twice daily	2 months	first stage of hypertension (SBP: 140–159 mmHg; DBP: 90–99 mmHg) sleep disorders	Changes in blood pressure

**Table 2:** Blood pressure data from the meta-analyzed studies.

First Author (year)	Duration	Group	Systolic Blood Pressure Before Treatment (Mean ± SD, mmHg; *p* Value)	Systolic Blood Pressure After Treatment (Mean ± SD, mmHg; *p* Value)	Diastolic Blood Pressure Before Treatment (Mean ± SD, mmHg; *p* Value)	Diastolic Blood Pressure After Treatment (Mean ± SD, mmHg; *p* Value)
Ann. F. Walker (2002) [17]	10 weeks	*Crataegus*	146.3 ± 6; N.A.	139.1 ± 5.5; N.A.	93.1 ± 3.8; N.A.	91.4 ± 4.6; N.A.
		placebo	154.5 ± 4.1; N.A.	139.6 ± 4.4; N.A	100 ± 4.5; N.A.	92.2 ± 3.7; N.A.
Asgary (2004) [18]	2 months	*Crataegus*	146 ± 16.23; 0.8	140 ± 3.49; 0.95	92 ± 6.03; 0.2	88 ± 2.49; 0.09
		placebo	148 ± 12.5; 0.8	140 ± 3.53; 0.95	95 ± 5.03; 0.2	90 ± 2.51; 0.09
	4 months	*Crataegus*	146 ± 16.23; 0.8	135 ± 6.95; 0.002	92 ± 6.03; 0.2	87 ± 3.74; 0.003
		placebo	148 ± 12.5; 0.8	142 ± 5.98; 0.002	95 ± 5.03; 0.2	91 ± 3.66; 0.003
Ann. F. Walker (2006) [19]	4 months	*Crataegus*	152.3 ± N.A.; 0.096	148.7 ± N.A.; 0.096	85.6 ± N.A.; 0.016	83 ± N.A.; 0.016
		placebo	147.4 ± N.A.; N.A.	146.6 ± N.A.; N.A.	84.5 ± N.A.; N.A.	85 ± N.A.; N.A.
Suzanna M. Zick (2009) [20]	6 months	*Crataegus*	109 ± 15; 0.25	106.9 ± 13.3; 0.26	66 ± 10; 0.76	65.2 ± 10.7; 0.45
		placebo	113 ± 19; 0.25	113.8 ± 13.2; 0.26	66 ± 10; 0.76	66.9 ± 11.4; 0.45
Al-Gareeb (2012) [15]	2 months	*Crataegus*	153.95 ± 3.14; N.A.	139.88 ± 2.81; N.A.	93.8 ± 2.19; N.A.	86.4 ± 2.54; N.A.
		placebo	153.08 ± 2.25; N.A.	148.64 ± 2.30; N.A.	92.11 ± 2.37; N.A.	90.88 ± 3.21; N.A.
	3 months	*Crataegus*	153.95 ± 3.14; N.A.	136.8 ± 3.32; N.A.	93.8 ± 2.19; N.A.	86.4 ± 1.92; N.A.
		placebo	153.08 ± 2.25; N.A.	150.34 ± 3.05; N.A.	92.11 ± 2.37; N.A.	91.38 ± 3.56; N.A.
Abbasi (2021) [21]	2 months	*Crataegus*	133.43 ± 6.5; N.A	118.14 ± 6.76; N.A.	90.71 ± 7.39; N.A.	77.14 ± 5.32; N.A.
		placebo	131.43 ± 6.73; N.A.	129.76 ± 8.28. N.A.	89.76 ± 4.32; N.A.	83.57 ± 6.73; N.A.

## Data Availability

Data sharing is not applicable.

## Abbreviations

The following abbreviations are used in this manuscript:

ACE	Angiotensin-converting enzyme
CAM	Complementary and alternative medicine
DBP	Diastolic blood pressure
FMD	Flow-mediated dilatation
MD	Mean difference
NO	Nitric oxide
NYHA	New York Heart Association
RCT	Randomized controlled trial
SBP	Systolic blood pressure
